# A novel genetic switch controls phase variable expression of CwpV, a *Clostridium difficile* cell wall protein

**DOI:** 10.1111/j.1365-2958.2009.06812.x

**Published:** 2009-08-06

**Authors:** Jenny E Emerson, Catherine B Reynolds, Robert P Fagan, Helen A Shaw, David Goulding, Neil F Fairweather

**Affiliations:** Division of Cell and Molecular Biology, Imperial College LondonLondon SW7 2AZ, UK

## Abstract

*Clostridium difficile* is a nosocomial pathogen that can cause severe gastrointestinal infections. *C. difficile* encodes a family of cell wall proteins, some of which are implicated in pathogenesis. Here we have characterized CwpV, the largest member of this family. CwpV is surface expressed and post-translationally processed in a manner analogous to the major S-layer protein SlpA. Expression of *cwpV* is phase variable, with approximately 5% of cells in a population expressing the protein under standard laboratory growth conditions. Upstream of *cwpV*, inverted repeats flank a 195 bp sequence which undergoes DNA inversion. Use of a *gusA* transcriptional reporter demonstrated that phase variation is mediated by DNA inversion; in one orientation *cwpV* is expressed while in the opposite orientation the gene is silent. The inversion region contains neither the promoter nor any of the open reading frame, therefore this system differs from previously described phase variation mechanisms. The *cwpV* promoter is located upstream of the inversion region and we propose a model of phase variation based on intrinsic terminator formation in the OFF transcript. A *C. difficile* site-specific recombinase able to catalyse the inversion has been identified.

## Introduction

*Clostridium difficile* is a Gram-positive, spore-forming anaerobe that causes a range of gastrointestinal diseases, ranging from diarrhoea to pseudomembraneous colitis, collectively termed *Clostridium difficile*-associated disease (CDAD) (Poxton *et al*., 2001; Bartlett, 2006). Infections in humans are most commonly associated with antibiotic therapy in nosocomial environments and have been increasing in both number and severity in recent years (Barbut *et al*., 2007). Antibiotics are thought to disrupt the normal intestinal flora, allowing *C. difficile* to colonize the gut. The principal virulence factors produced by *C. difficile* are two cytotoxins, TcdA and TcdB. The modes of action of TcdA and TcdB are well described: both toxins, which are highly related in structure and function, are glucosyltransferases that target small GTPases resulting in alterations in the cytoskeleton, apoptosis, infiltration of neutrophils and damage to the gut mucosa (Just *et al*., 1995; Voth and Ballard, 2005).

In order to colonize their hosts, pathogenic bacteria must both evade the immune response and interact with host cells, often adhering to specific surface-localized molecules (Pizarro-Cerda and Cossart, 2006). Bacterial surface proteins and structures play key roles in these processes. In *C. difficile* the major surface proteins are within the S-layer, a paracrystalline proteinaceous array that completely coats the bacterium. The S-layer is formed of two proteins, the high-molecular-weight S-layer protein (HMW SLP) and the low-molecular-weight (LMW) SLP, which are products of the SlpA precursor (Calabi *et al*., 2001). After cleavage of the SlpA precursor, the resulting HMW and LMW SLPs interact via defined domains to form a stable heteromeric complex (Fagan *et al*., 2009). In the genome of *C. difficile* 630, 28 paralogues of the HMW SLP have been identified (Sebaihia *et al*., 2006), and transcriptomic and proteomic studies have shown several of these proteins to be expressed under laboratory conditions (Wright *et al*., 2005; Emerson *et al*., 2008). Antibodies against some of these proteins are found in serum of CDAD patients (Wright *et al*., 2008) implying at least some of this family of cell wall proteins (CWPs) are expressed *in vivo* during infection. All of these CWPs contain two or three cell wall binding motifs (Pfam PF04122) in addition to a unique domain that is proposed to specify function.

In *C. difficile* several CWPs have been identified that may interact with the host to facilitate adherence. These include the adhesin Cwp66 (Waligora *et al*., 2001), the fibronectin binding protein Fbp (Hennequin *et al*., 2003) and the protease Cwp84 (Janoir *et al*., 2007). The HMW SLP also shows strong binding to the intestinal epithelium of both human and mouse and anti-HMW SLP antibodies block adherence to HEp-2 cells (Calabi *et al*., 2002). SLPs have been shown to induce inflammatory and regulatory cytokines in human monocytes and dendritic cells, suggesting that during infection they may interact with the immune system (Ausiello *et al*., 2006).

In this study we investigate the expression, regulation and processing of another member of the CWP family, which we term CwpV. We show that CwpV is surface expressed and post-translationally processed in a manner analogous to SlpA. We demonstrate that *cwpV* expression is phase variable and that the mechanism of transcriptional control differs from those previously described. We propose a mechanism for this phase variation and have identified one *C. difficile* site-specific recombinase that mediates DNA inversion.

## Results

### Surface localization and processing of CwpV

**Fig. 1:**
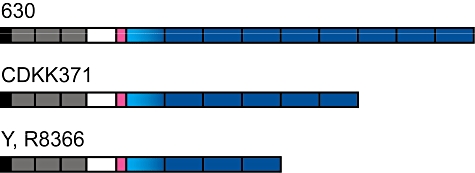
Domain architecture of CwpV. The N-terminus of CwpV contains a signal sequence (black), followed by three PF04122 cell wall anchoring motifs (grey) and a region of unknown function (white). A serine–glycine rich region (pink) precedes a number of repeats (blue). The repeats are almost identical in sequence with the exception of the N-terminal repeat which shows more sequence diversity. Different strains have different numbers of repeats. Sequences were deposited at EMBL with accession numbers FM17250, FM17252 and FM17254.

**Fig. 2:**
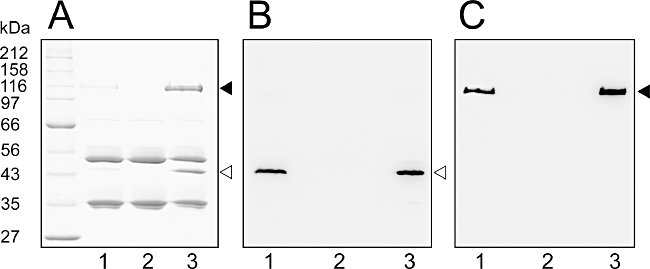
Surface expression of CwpV. *C. difficile* strains were grown overnight in BHI broth. S-layer extracts were prepared and analysed by SDS-PAGE and Western blotting. A. Coomassie blue-stained gel. B. Western blot using anti-CwpVNter (1:5000). C. Western blot using anti-CwpVrpt1 (1:5000). Lane 1: 630; lane 2: 630Δ*erm*Δ*cwpV*; lane 3: 630Δ*erm*Δ*cwpV*(pCBR044). The 40 kDa (◃) and 120 kDa (◂) fragments of CwpV are indicated. For Western blots the total protein loaded in lane 3 was fivefold less than in lanes 1 and 2 to allow all bands to be visualized using one exposure time.

In *C. difficile* 630, the *cwpV* gene (CD0514) encodes a predicted protein of 167 kDa, containing an N-terminal signal peptide, three PF04122 cell wall binding motifs presumed to mediate attachment to the underlying cell wall, a serine/glycine-rich region and finally nine repeats, each of 120 amino acids (Fig. 1). Analysis of other strains of *C. difficile* revealed that the number of repeats is variable, with CDKK371 having six repeats and R8366 and Y having four repeats. CwpV is annotated as a putative adhesin (Sebaihia *et al*., 2006) based on homology to a known haemagglutinin from *Salmonella typhimurium*. To further characterize *cwpV,* the gene from *C. difficile* 630 was cloned into pMTL960, an *Escherichia coli*–*C. difficile* shuttle vector. The promoter P_*cwp2*_ utilized was that of another cell wall protein, Cwp2, that is moderately expressed in *C. difficile* (Calabi and Fairweather, 2002). We also constructed a *cwpV* gene knockout in *C. difficile* 630Δ*erm* using the Clostron technique (Heap *et al*., 2007). Expression of CwpV was investigated using two antibodies; one raised against the N-terminal cell wall binding domain (anti-CwpVNter) and a second raised against the first of the nine repeats (anti-CwpVrpt1). Cell wall extracts of *C. difficile* were prepared using low pH glycine extraction, which enriches for the SLPs and other minor surface localized proteins including CwpV (Calabi *et al*., 2001; Wright *et al*., 2005). Figure 2 shows that in strain 630, CwpV is detected as two fragments. The smaller 40 kDa fragment represents the cell wall binding domain and reacts with anti-CwpVNter. The larger 120 kDa fragment contains the repeat sequences and reacts with anti-CwpVrpt1. This indicates that CwpV is post-translationally cleaved in a manner analogous to the major S-layer protein SlpA (Calabi *et al*., 2001). Neither fragment is observed in the *cwpV* deletion strain 630Δ*erm*Δ*cwpV*. In the complemented mutant 630Δ*erm*Δ*cwpV*(pCBR044) expression is clearly at a higher level than the wild-type strain, as together both fragments represent a considerable percentage of the total protein in the glycine extract. Full-length CwpV was never observed either in cell wall extracts or in whole cell lysates (data not shown), even in the complemented mutant, indicating that processing is highly efficient and that it may be required for correct localization of the protein in the cell wall.

### Expression of CwpV is phase variable

**Fig. 3:**
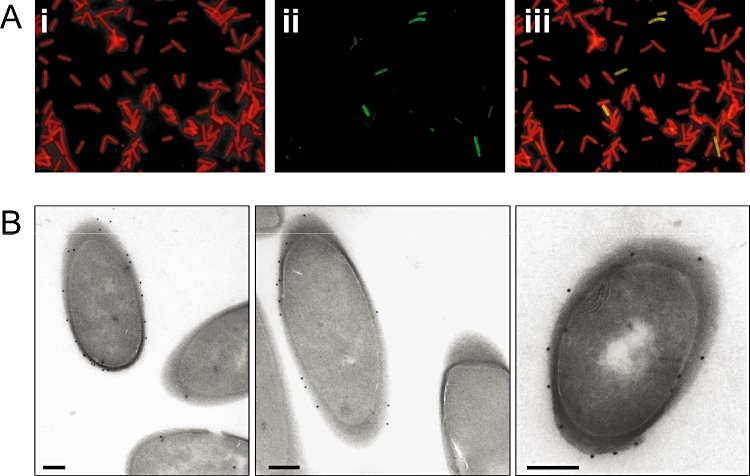
Phase variable expression of CwpV. A. *C. difficile* 630 were grown in BHI broth and labelled with (i) rat anti-LMW SLP (red) showing that all bacteria are surface labelled and (ii) anti-CwpVrpt1 (green) showing a fraction of bacteria labelled. Labelling was visualized using Texas Red anti-rat conjugate and fluorescein anti-rabbit conjugate. (iii) merge of (i) and (ii). B. Immunogold electron microscopy of 630 labelled with anti-CwpVrpt1 antibodies. Staining is seen only on the external surface of cells. Bar = 200 nm.

Localization of CwpV in 630 was investigated by immunofluorescence on intact bacteria using anti-CwpVrpt1 and co-staining with anti-LMW SLP antibody. All bacteria were labelled with anti-LMW SLP whereas only a small proportion of cells were stained with anti-CwpVrpt1 (Fig. 3A). In those bacteria that did express CwpV, the protein was localized to the cell surface as observed by immunogold electron microscopy (Fig. 3B). In cultures grown in brain–heart infusion (BHI) broth the proportion of cells expressing CwpV was found to be consistently between 5% and 10%. A similar proportion of CwpV positive cells was seen in colonies grown on BHI agar and we were unable to detect single colonies where all the cells were either expressing or not expressing CwpV (data not shown).

**Fig. 4:**
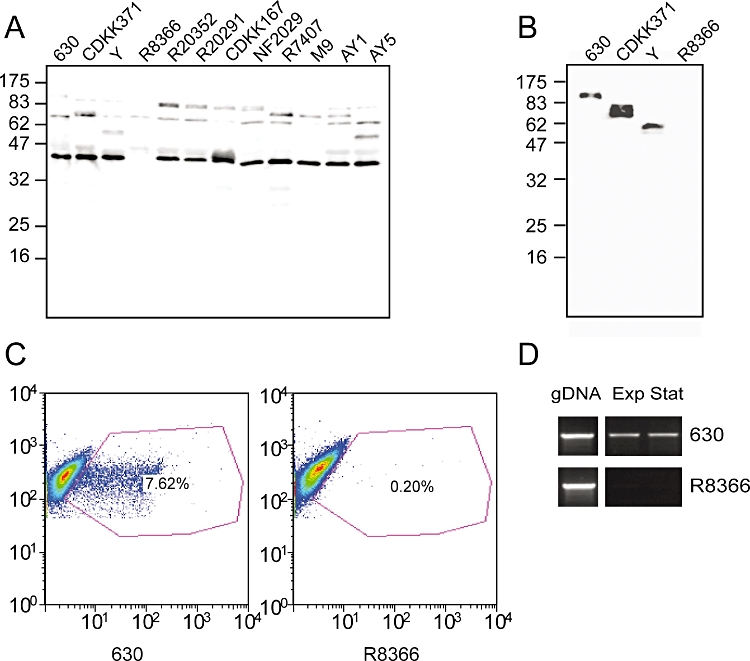
Expression of *cwpV* is seen in all strains except R8366. A. Western blot analysis of a panel of *C. difficile* strains. Glycine extracts were prepared and probed with anti-CwpVNter antibody (1:1000). A band at 40 kDa was observed in all strains, with the exception of R8366. B. Western blot analysis of stains using anti-CwpVrpt1 antibody. The sizes of bands (120 kDa in 630, 82 kDa in CDKK371 and 57 kDa in Y) are consistent with the predicted sizes of the repetitive regions of these strains. R8366 showed no reaction. C. Flow cytometry analysis of 630 and R8366 stained using anti-CwpVrpt1 antibody and fluorescein anti-rabbit conjugate. Side scatter, which is representative of particle size, is shown on the *y*-axis and fluorescence intensity on the *x*-axis. Frequency of events (bacteria) is indicated by colour. Unstained bacteria are visualized in a grouping at the left of the graph and the purple polygon represents the area in which events were scored as positive. 7.62% of 630 bacteria are positively stained, whereas negligible positive events are seen with R8366. D. RNA was extracted from mid-exponential (Exp) and early stationary (Stat) phase cultures, reverse transcribed and the conserved 5′ region of *cwpV* was amplified by PCR using primers NF654 and NF655. Transcription is seen in 630 but not in R8366. Genomic DNA (gDNA) was used a positive control.

Expression of CwpV was analysed in a panel of strains using Western blotting with the anti-CwpVNter antibody. All strains analysed were found to express CwpV with the exception of R8366 (Fig. 4A). Representative strains encoding four, six and nine copies of the 120 bp repeat in their genome were further investigated using anti-CwpVrpt1 antibody. All these strains, with the exception of R8366, expressed this domain with a detected band size representative of the number of encoded repeats (Fig. 4B). Flow cytometry analysis of 630 and R8366 using anti-CwpVrpt1 antibodies revealed approximately 7% of 630 cells to express CwpV, whereas R8366 cells were not labelled (Fig. 4C). DNA sequence analysis revealed that R8366 contained an intact *cwpV* gene (data not shown), suggesting that the expression defect might be at the level of transcription. Reverse transcription polymerase chain reaction (PCR) analysis of 630 and R8366 showed that transcripts were detected in 630 grown to exponential and stationary phase, whereas no transcripts were detected in R8366 (Fig. 4D).

### Inverted repeats upstream of *cwpV* mediate DNA inversion

**Fig. 5:**
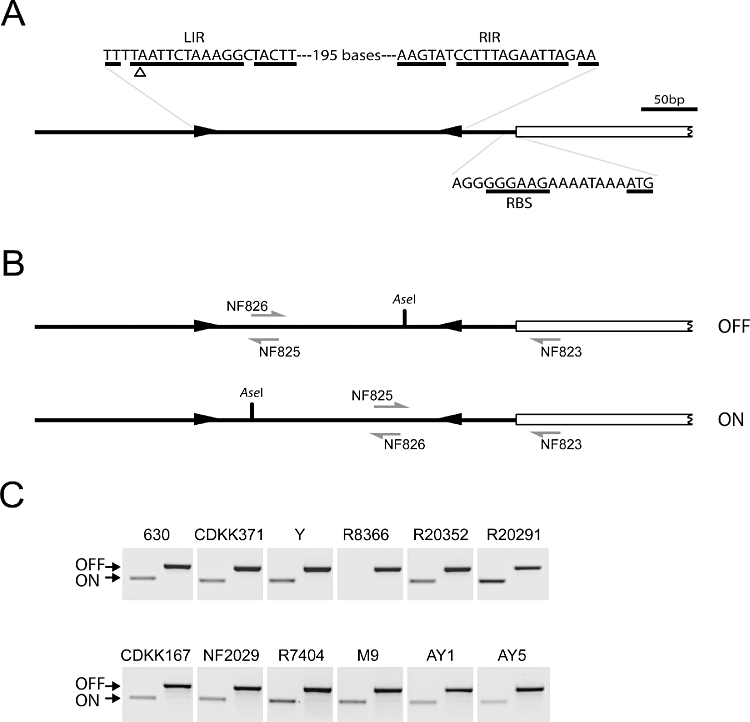
The region upstream region of cwpV contains inverted repeats and undergoes DNA inversion. A. Diagrammatic representation of features upstream of *cwpV* in 630. Two inverted repeats, LIR and RIR, are found upstream of the *cwpV* translational start site in the genomic DNA. DNA is shown in the published OFF orientation and the two base pair deletion in strain R8366 is indicated (Δ). The predicted RBS sequence and the ATG are shown. B. Diagrammatic representation of the inversion region showing the two orientations. The annealing sites of primers used to probe the orientation are shown. NF823 anneals outside the inverted repeats. NF825 and NF826 anneal inside the inversion region in opposite orientations. C. PCR amplification to detect the orientation of the inverted repeats in genomic DNA. Two PCR reactions were performed on genomic DNA from each strain of *C. difficil*e to determine the orientation of the inversion region. NF823 and NF825 give a product in the ON orientation; NF823 and NF826 give a product in the OFF orientation. All strains were found to contain the DNA switch in both orientations apart from strain R8366 in which no DNA in the ON orientation can be detected. DNA sequencing of genomic DNA of 630, R20352 and R20928 and several other strains revealed the majority of DNA is in the OFF orientation.

Examination of the DNA sequence upstream of the *cwpV* open reading frame (ORF) revealed a pair of imperfect 21 bp inverted repeats (IRs) 57 bp upstream of the start codon and separated by 195 bp (Fig. 5A). A small number of previously described genetic switches in bacteria rely on DNA inversion catalysed by site-specific recombinases, which bind to IRs and catalyse strand exchange. The best-characterized example of such a system is the phase variation of Type 1 fimbriae in *E. coli*, where the *fimA* promoter is located between two IRs (Abraham *et al*., 1985). Inversion of the sequence bounded by the IRs, which is mediated by specific recombinases FimB and FimE, results in ON–OFF switching of expression of *fimA* (reviewed in Henderson *et al*., 1999).

In order to determine whether the IRs upstream of *cwpV* lead to inversion of the DNA sequence between the IRs, PCR was performed on genomic DNA using primer NF823 with either NF826, designed to detect the orientation as published in the genome sequence (Sebaihia *et al*., 2006) (hereafter referred to as OFF), or with NF825, designed to detect the inverted orientation (ON) (Fig. 5B). As shown in Fig. 5C, bands were detected with both primer pairs in all strains tested apart from R8366, indicating that the genomic DNA between the IRs of these strains does undergo inversion. DNA sequencing of these PCR products revealed that recombination occurs within the central region of identity of the IR as the outer T/G mismatch does not differ between the ON and OFF sequences, whereas the inner C/T mismatch inverts along with the inter-IR DNA (data not shown).

In strain R8366, which does not express CwpV, DNA could only be detected in the OFF orientation. This suggests DNA inversion is greatly reduced or absent in this strain. Examination of the DNA sequence upstream of *cwpV* in a number of strains revealed very high sequence conservation (data not shown). However, in R8366, a two-nucleotide deletion was observed in the left IR (LIR) (Fig. 5A). We therefore hypothesize that this deletion prevents DNA inversion in strain R8366 and that inversion is necessary for expression of *cwpV*.

### Initiation of transcription is upstream of the IRs

**Fig. 6:**
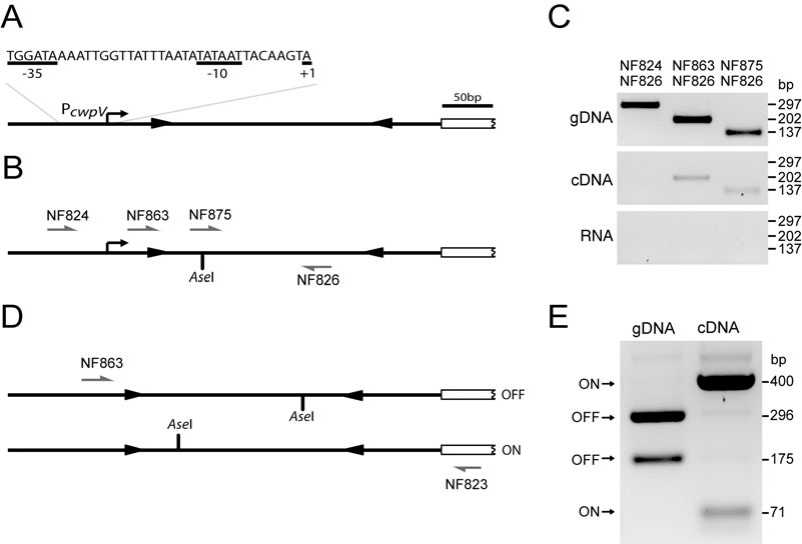
Transcription of *cwpV* in *C. difficile*. A. Diagrammatic representation of the *cwpV* promoter and transcriptional start site as determined by 5′ RACE. B and C. PCR amplification to confirm the transcriptional start site. cDNA was prepared from 630 and amplified by PCR using primer NF826 together with primer NF824, NF863 or NF875. Amplification is seen with primers NF875 and NF863 but not NF824, confirming that the start site of transcription lies between the NF824 and NF863 binding sites. Positive (gDNA) and negative (RNA) controls behaved as expected. D and E. RFLP analysis of *cwpV* cDNA. cDNA was prepared from 630, amplified by PCR using primers NF863 and NF823 and digested with *Ase*I. Bands detected from DNA in the ON and OFF orientations are indicated. Densitometry analysis revealed *cwpV* mRNA is predominantly in the ON orientation (99%), and the genomic DNA (gDNA) is predominantly in the OFF orientation (96%).

In order to locate the start site of transcription of *cwpV* we performed 5′ RACE (Rapid Amplification of cDNA Ends) analysis on mRNA isolated from strain 630. Several clones were obtained and were sequenced to determine the 5′ nucleotide of the transcript. Surprisingly, the 5′ end was not present within the sequence bounded by the IRs, but was localized 36 or 37 bases upstream of the LIR (data not shown; Fig. 6A). Putative −10 and −35 sequences, closely matching those recognized by the major bacterial sigma factor σ70 in other Gram-positive promoters (Wosten, 1998), were identified upstream of the site of initiation (see Fig. 6A). A second subset of clones revealed a start site four bases upstream of the LIR, but no −35 or −10 sequences are seen upstream of this site. It is therefore likely that these transcripts result from trimming of a longer transcript rather than the existence of two transcriptional start sites. To further confirm the location of the transcriptional start site of *cwpV*, cDNA was prepared from 630 and PCR-amplified using a reverse primer (NF826) located within the IRs (in the ON orientation) with a series of forward primers localized further upstream (Fig. 6B). Products were successfully amplified using forward primers NF863 or NF875 located within and downstream of the LIR respectively, but not with primer NF824 located further upstream (Fig. 6C). These results confirm that the promoter of *cwpV* (P_*cwpV*_) is located proximal to and upstream of the LIR.

Interestingly, all RACE clones sequenced were observed to be in the ON orientation; no clones were isolated that contained the sequence between the IRs in the OFF orientation. To further probe the role of DNA inversion in expression of *cwpV*, RNA was extracted from 630, converted to cDNA and PCR-RFLP analysis was carried out. Using primers NF863 and NF823 a product was amplified extending from the observed transcriptional start site to within the *cwpV* gene. This PCR product was then digested with *Ase*I, the cleavage site of which is situated asymmetrically within the region bounded by the IRs (Fig. 6D). The results (Fig. 6E) clearly show that the *cwpV* mRNA is almost entirely present in the ON orientation. In contrast, the majority of the genomic DNA is present in the OFF orientation. It therefore appears that stable full-length transcripts can only be produced from genomic DNA in the ON orientation. This is consistent with the detection of CwpV expression in only around 5% of cells, corresponding to those few cells with genomic DNA in the ON orientation. The region of DNA bounded by the IRs, located between the promoter and the *cwpV* ORF, was named the *cwpV* switch.

### Characterization of the *cwpV* genetic switch using a β-glucuronidase reporter

**Fig. 7:**
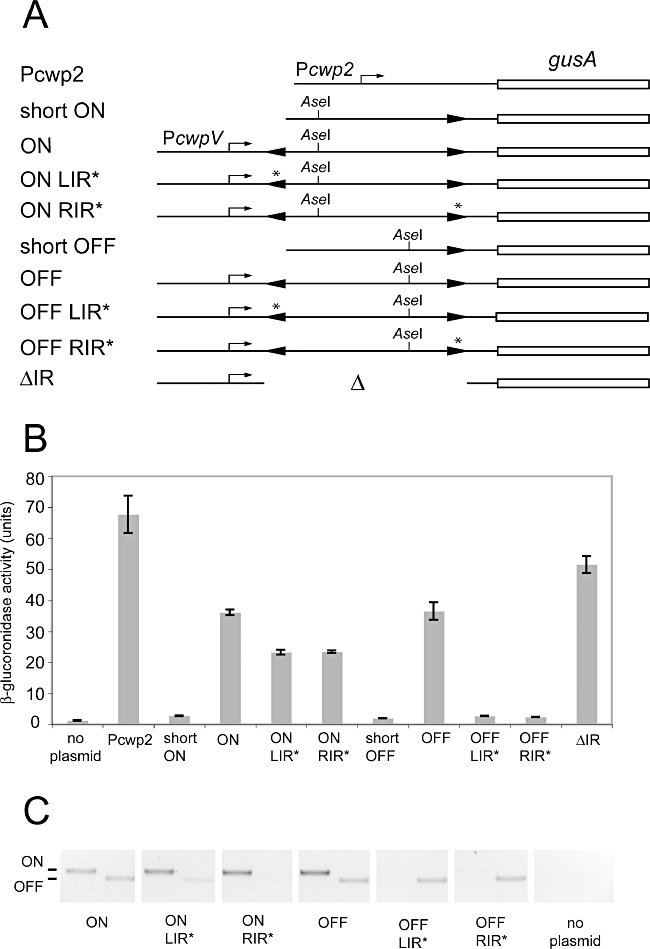
Analysis of transcription from P_cwpV_ in *C. difficile* using a GusA reporter. *C. difficile* 630 containing plasmids with different components of the *cwpV* switch upstream of *gusA* were grown in liquid medium, harvested and the β-glucoronidase activity measured. A. Diagrammatic representation of the promoter regions present in the plasmids used in this experiment. Plasmids used were: P_*cwp2*_*,* pCBR034; short ON, pCBR035; ON, pCBR037; ON LIR*, pCBR039; ON RIR*, pCBR041; short OFF, pCBR036; OFF, pCBR038; OFF LIR*, pCBR040; OFF RIR*, pCBR042; ΔIR, pCBR043. B. Enzyme activities of cell lysates. Error bars represent the standard deviations (*n* = 3). C. PCR analysis of DNA isolated from cultures using primers to detect the ON (left) (NF793 and 826) and OFF (NF793 and 825) orientations of the *cwpV* switch.

To study further the ability of the *cwpV* switch to control transcription, a series of transcriptional reporter constructs was created in *E. coli* using *gusA*, encoding β-glucuronidase, which has been used previously to measure activity of *C. difficile* promoters (Mani and Dupuy, 2001). The promoterless *gusA* gene together with the *tcdB* ribosome binding site from pTUM177 was first cloned into pUC19 downstream of P_*cwp2*_. GusA expression driven by P_*cwp2*_ could then act as a positive control for the reporter system. DNA fragments located upstream of *cwpV* were then amplified from 630 genomic DNA and cloned upstream of *gusA* in order to assay their ability to drive expression. Two different lengths of fragment were used: (i) long fragments containing DNA extending 432 bp upstream of the start codon of *cwpV* and including P_*cwpV*_; (ii) short fragments which extended 273 bp upstream of the start codon of *cwpV* and did not include the LIR. Plasmids with the long upstream sequence were also constructed containing a 2 bp deletion in the LIR (LIR*) or right IR (RIR*), as found in the LIR of R8366 which does not undergo DNA inversion. All constructs were generated such that the *cwpV* switch was in each of the two orientations, termed ON and OFF. This was possible because the *cwpV* switch does not invert in *E. coli* (see below). A diagrammatic representation of the different constructs is shown in Fig. 7A.

Once constructed in pUC19 the promoter*-gusA* fragments were then subcloned into the *E. coli*–*C. difficile* shuttle vector pMTL960 (Purdy *et al*., 2002) and transferred to *C. difficile* 630 by conjugation. Exponential cultures were analysed for GusA activity. As expected, the plasmid containing the long upstream sequence in the ON orientation (pCBR037) was able to drive expression of *gusA* (Fig. 7B). However, expression was also observed from the long OFF plasmid (pCBR038). PCR analysis of the plasmids with long upstream sequences after growth in *C. difficile* confirmed that both originally ON and originally OFF plasmids contained DNA in both the ON and OFF orientations (Fig. 7C). This suggests that in *C. difficile* the *cwpV* switch on this plasmid can undergo inversion, resulting in sufficient levels of the switch in the ON orientation to allow expression of *gusA*.

Strains containing LIR* or RIR* mutant derivatives of the ON plasmids (pCBR039 and pCBR041) also expressed *gusA*, whereas those in the OFF orientation (pCBR040 and pCBR042) did not. PCR analysis showed that OFF plasmids with the LIR* and RIR* mutations contained DNA only in the OFF orientation, confirming that the 2 bp deletion did indeed lock the DNA in one orientation and prevent switching. This is consistent with a complete lack of GusA activity detected in these strains and a lack of expression of CwpV in strain R8366, which contains the same upstream sequence as OFF LIR*. DNA in the ON orientation and containing the 2 bp deletion in either IR drove expression of GusA, indicating that the deletion itself does not prevent expression. Therefore it is the inability to switch that causes the phenotype of strain R8366. PCR analysis of ON LIR* detected some of the *cwpV* switch in the OFF orientation showing that the orientation of ON LIR* does not remain fully locked ON (Fig. 7C), suggesting that this 2 bp deletion inhibits the OFF to ON inversion more effectively than ON to OFF.

Plasmids containing the short fragment of DNA upstream of *cwpV* (pCBR035 and pCBR036) did not drive the expression of *gusA*, regardless of the orientation of the *cwpV* switch, consistent with the promoter being located upstream of the LIR. Interestingly, deletion of the entire *cwpV* switch region (pCBR043) resulted in expression of *gusA*, demonstrating that the *cwpV* promoter functions in the absence of the *cwpV* switch. This indicates that the *cwpV* switch is a negative regulator of expression when present in the OFF orientation.

### A model for the *cwpV* switch mechanism

Considering the mechanisms whereby DNA inversion could act as a negative regulator of *cwpV* expression, two possibilities seem likely. First, one or more proteins could bind to the DNA or RNA in the OFF orientation and prevent transcription or translation. Second, intrinsic properties of the DNA or RNA in the OFF orientation could prevent transcription or translation. These two mechanisms are not mutually exclusive as there may be a specific structure in the OFF DNA/RNA to which protein binding could mediate the negative regulation.

**Fig. 8:**
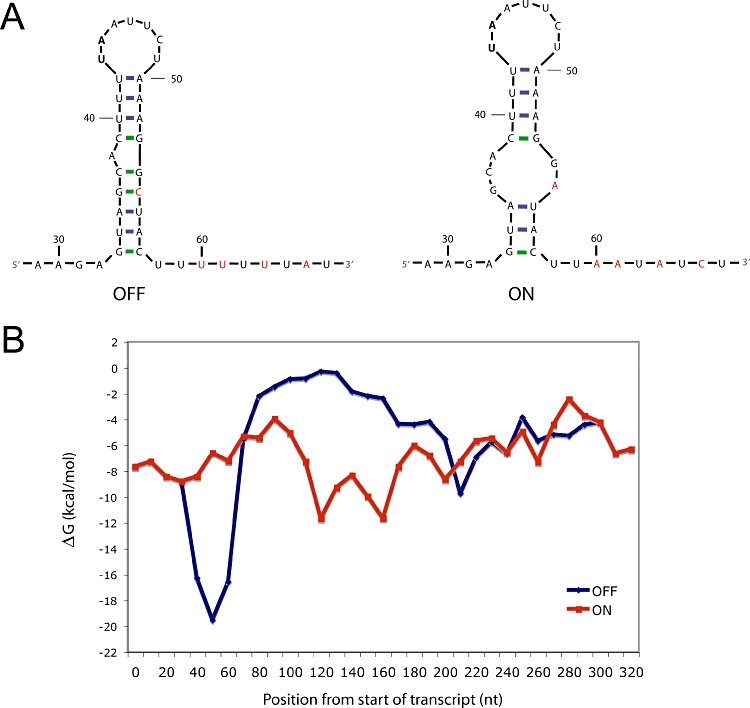
RNA structure and free energy predictions of the *cwpV* DNA switch. A. Predicted RNA structures found at the junction between the LIR and the region of inversion. Nucleotides that differ between the two orientations are shown in red. GC base pairing is shown in green and AU base pairing in blue. Numbering is from the predicted transcriptional start site. The two bases in bold are those deleted in strain R8366. B. Free energy predictions for the two orientations, measuring the ΔG of 60 nt regions and sequentially moving along the sequence in 10 nt segments. Data were obtained using the on-line program *mfold* (Zuker, 2003). The free energy peak seen at 50 nt in the OFF orientation corresponds to the predicted RNA structure shown in A.

To explore the second hypothesis and search for the presence of intrinsic terminators, the RNA structure of transcripts from the two orientations were predicted using *mfold*, a program that predicts the structure and free energies of RNA sequences (Zuker, 2003). A stable stem loop structure consisting of a 9 bp stem containing four G-C bp was predicted to form in the OFF orientation at the junction between the LIR and the region of inversion (Fig. 8A). This stem loop structure is followed by a poly-U tract. In the ON orientation the sequence of this region is altered by a number of base pairs (coloured red in Fig. 8A) due to inversion of the *cwpV* switch and the predicted RNA structure is therefore different. A model for predicting intrinsic terminators in *E. coli* has been developed (Lesnik *et al*., 2001) and has been used to predict intrinsic terminators in a number of bacterial species including *Clostridium acetobutylicum* (Paredes *et al*., 2004). This model is built from sequence constraints based on known terminator sequences from *E. coli*. Briefly there must be a stem of 4–18 bp, containing at least four G-C bp, a loop of 3–10 nt and following the stem a poly-U tract with sufficient U-content. Quantitative models explaining how such structures destabilize the transcription complex have been reported (Yager and von Hippel, 1991). The structure predicted for the OFF orientation fulfils all the criteria for an intrinsic terminator, whereas the ON stem only contains two G-C bp and has a large internal loop and therefore does not fulfil the criteria. To further compare the stability of predicted RNA structures of the ON and OFF transcripts, quantitative predictions of free energy of RNA folding for the transcript in the two orientations were calculated for 60 nt long stretches of sequence, systematically moving along the transcript in 10 nt jumps. A peak in free energy, characteristic of an intrinsic terminator (Washio *et al*., 1998), is seen in the OFF orientation at the junction between the LIR and the inversion region; this peak is not present in the ON orientation (Fig. 8B). Based on these predictions our current hypothesis for the mechanism underlying phase variation of *cwpV* is that transcription initiates upstream of the LIR regardless of the orientation of the *cwpV* switch, but in the OFF orientation transcription is terminated upon reaching the region of inversion due to an intrinsic terminator. In the ON orientation, transcription can continue through the inversion region to the ORF and produce a full-length transcript.

### Inversion of the *cwpV* switch does not occur in *E. coli* or *Clostridium perfringens*

**Fig. 9:**
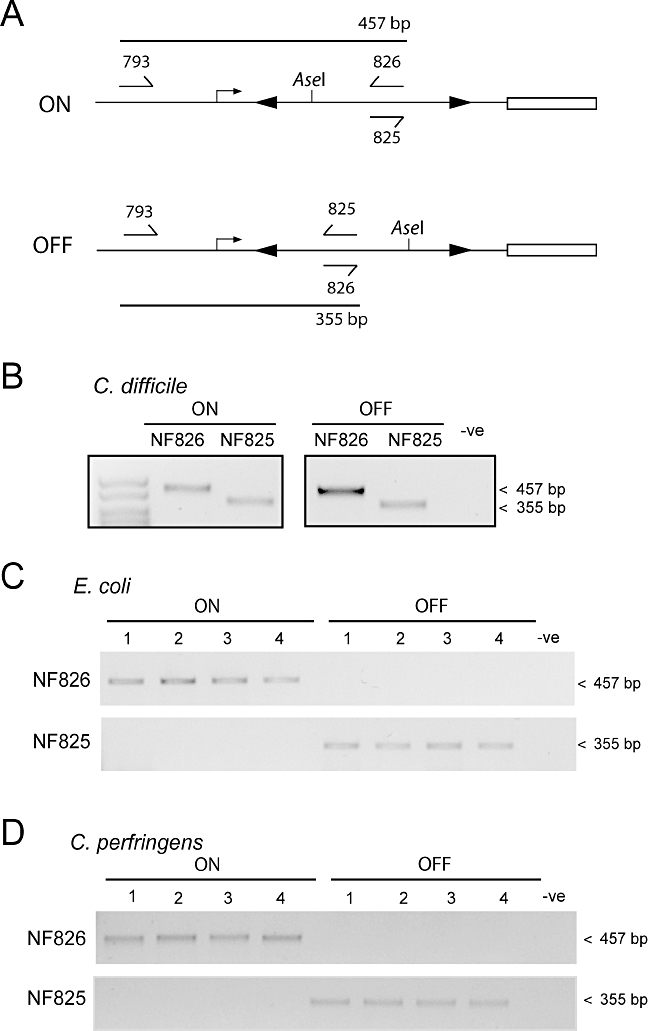
DNA inversion is specific to *C. difficile* and does not occur in *C. perfringens* or *E. coli*. Orientation-specific PCRs were carried out to determine whether DNA inversion of the *cwpV* switch occurs in *E. coli* or *C. perfringens*. A. The *cwpV* switch region in the ON and OFF orientations, in pCRB037 and pCRB038, respectively, showing the position of the primers used. B. PCR products of the ON and OFF plasmids after introduction into *C. difficile*. DNA inversion occurs in each case as seen by the appearance of both PCR products. C and D. The plasmids were introduced into *E. coli* (C) and *C. perfringens* (D). Four independent transformants or transconjugants were picked and in each case no DNA inversion is seen.

Our results suggest that one or more site-specific recombinases in *C. difficile* mediate DNA inversion to control expression of *cwpV*. The ability of different species to mediate inversion of the *cwpV* switch was assessed by PCR analysis of plasmids pCBR037 and pCBR038. These plasmids contain the *cwpV* switch in the ON and OFF orientations respectively. The orientation of the *cwpV* switch can be determined by PCR using primers which anneal to opposite strands within the region of inversion, paired with a primer outside the region of inversion (see Fig. 9A). In *C. difficile*, inversion of the *cwpV* switch occurred in both the ON and OFF plasmids, as shown by the appearance of PCR products using either primer NF825 or NF826 (Fig. 9B). In contrast, in *E. coli* (Fig. 9C) or *C. perfringens* (Fig. 9D), no inversion was seen in four independent isolates containing either plasmid. Therefore it is likely that *C. difficile* encodes one or more specific recombinases which catalyse the inversion of the *cwpV* switch and these enzymes are not present in *C. perfringens* or *E. coli*.

### Identification of a *C. difficile* recombinase mediating the *cwpV* switch

**Fig. 10:**
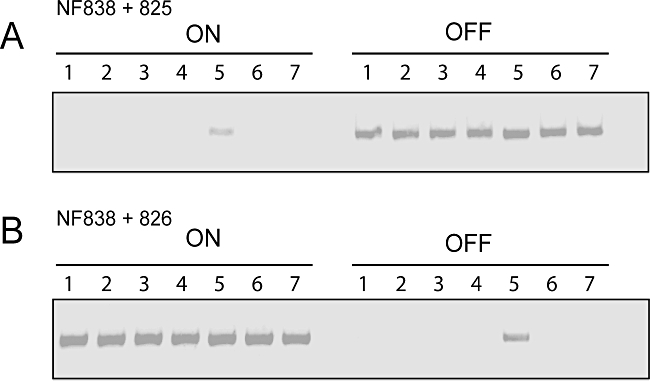
Identification of a recombinase mediating DNA inversion of the *cwpV* switch. Genes for seven recombinases (*CD1932, CD1905, CD2066, CD1822, CD1167, CD3578* and *CD1222*) were cloned into pACYCDuet-1 and co-transformed into *E. coli* BL21λDE3 with pUC19 plasmids carrying the *cwpV* switch in either the ON or OFF orientations (pCBR026 or pCBR027). PCR reactions with primer pairs NF838 and NF825 (A) and NF828 and NF826 (B) were then carried out. Recombinase 5 (*CD1167*) mediated inversion in both the ON to OFF and OFF to ON orientations.

A search the *C. difficile* 630 genome identified 22 putative recombinases. However, 16 of these are associated with the many mobile genetic elements (MGEs) in this strain, and are largely absent from other strains as determined by microarray analysis (Stabler *et al*., 2006). All *C. difficile* strains analysed to date exhibit *cwpV* switch inversion (except R8366), making it likely that the site-specific recombinase(s) responsible for inversion are well conserved. A total of seven putative site-specific recombinase genes were chosen for analysis: three of the best-conserved MGE-associated recombinases, one putative housekeeping recombinase and all three non-MGE-associated recombinase genes. The genes were each cloned into pACYCDuet-1 to allow their expression as His-tagged proteins, and then transformed into *E. coli* BL21λDE3 containing pUC19-based plasmids with the *cwpV* switch in either the ON or OFF orientation (pCBR026 or pCBR027). Orientation-specific PCR showed that only one recombinase, CD1167, mediated DNA inversion (Fig. 10). This gene, which we designate *recV*, is a tyrosine recombinase of the XerC/XerD family of DNA recombinases and can catalyse both ON to OFF and OFF to ON inversion. None of the other recombinases tested catalysed DNA inversion in *E. coli*. Western blotting using anti-His tag antibodies verified that all recombinases were expressed in *E. coli*, producing bands of the expected size (data not shown).

## Discussion

In this study we describe the phase variation of *cwpV*, a gene encoding a cell surface protein of *C. difficile*. Phase variation mediated by DNA inversion has been described in a small number of bacterial systems (van der Woude and Baumler, 2004). The best-characterized systems involve inversion of a promoter or part of the ORF. In the former, a promoter in one orientation actively transcribes a gene whereas in the opposite orientation either no gene transcription occurs or an alternative gene is transcribed. For example, in the *E. coli fim* system, the *fimA* gene product is either expressed or not, depending on the promoter orientation (Abraham *et al*., 1985). In the *Salmonella hin* system, in one orientation the promoter directs expression of the H1 flagellin, whereas in the opposite orientation it directs expression of H2 flagellin (Zieg *et al*., 1977). In both these systems the recombinase(s) involved in DNA inversion are located either within the inverted DNA (*S. typhimurium hin*) or within the immediate vicinity of the switch (*E. coli fim*) (reviewed in van der Woude and Baumler, 2004). It is interesting that *recV* is not located in the vicinity of *cwpV*. This fact, along with the presence of the *cwpV* switch and *recV* in all *C. difficile* strains analysed within a diverse set of isolates, suggests that this switch and *recV* have not been recently acquired by horizontal gene transfer.

In the case of *cwpV*, we have established that the promoter and transcriptional initiation site are situated upstream of the *cwpV* switch and that DNA inversion controls the expression of *cwpV.* The production of stable full-length *cwpV* transcripts is only possible from template DNA in the ON orientation. This mechanism for controlling gene expression involving DNA inversion encompassing neither the promoter nor the ORF is to our knowledge completely novel. Furthermore, by using a *gusA* transcriptional reporter in *C. difficile*, we have shown that the *cwpV* switch region is not required for expression from the *cwpV* promoter, and that the *cwpV* switch must therefore act *in cis* to negatively regulate expression. We propose a model to account for these observations based on predicted secondary structure of the mRNA transcripts. This mechanism involving transcriptional termination modulated by DNA inversion is to our knowledge unique. Transcripts in the OFF orientation are predicted to form a stable stem loop structure followed by a poly-U tract, which induces transcriptional termination at some 60–70 nucleotides downstream of the transcript initiation site. In the ON orientation the predicted structure does not fulfil the criteria for an intrinsic terminator and transcription can proceed unhindered. We acknowledge the possibility that, in the OFF orientation, transcription could also be affected by binding a repressor molecule or that control could be mediated by a combination of these mechanisms.

Interestingly modulation of transcription termination is also seen in the *E. coli fim* system, where a Rho-dependent phase variable transcriptional terminator is present downstream of the *fimE* recombinase gene in phase OFF cells (Joyce and Dorman, 2002; Hinde *et al*., 2005). In phase ON bacteria, termination of the *fimE* transcript does not occur and the mRNA transcript is longer and more stable, leading to increased levels of FimE recombinase and biasing the switch to the phase OFF. However, in the *fim* switch, the primary level control of *fimA* expression is inversion of the promoter, whereas in the *cwpV* switch described here, transcription termination is the key event determining expression of *cwpV*.

A common theory to account for phase variation in bacterial pathogens is that alteration of surface structures allows evasion of the immune responses. Other explanations include modulation of adhesin expression that could facilitate detachment of bacteria from host substrates, resulting in dissemination of the bacteria from the host. However, in many cases the biological significance of phase variation remains a mystery (van der Woude, 2006). To date we have been unable to define a functional role for CwpV. Although annotated as a haemagglutinin (Sebaihia *et al*., 2007), we have not found haemagglutination activity despite using blood from a number of species (data not shown). The presence of multiple repeats in a surface protein of a bacterial pathogen usually suggests adhesin activity, but to date we have been unable to define such a role for CwpV. It is possible, however, that CwpV does mediate either bacteria–host or bacteria–bacteria interactions, and this is currently under investigation.

For *C. difficile*, survival in the enteric system may require adaptation to changing environmental conditions, and the regulated expression of *cwpV* may reflect responses that allow adaption to particular niches during infection. In the enteric symbiont *Bacterioides fragilis*, DNA inversion is known to operate on a large scale, controlling multiple loci including biosynthetic operons for surface localized capsular polysaccharides (Patrick *et al*., 2003; Fletcher *et al*., 2007). Mutagenesis studies have demonstrated that *B. fragilis* requires the capacity to produce a repertoire of capsular polysaccharides and that mutants expressing a single polysaccharide are defective in intestinal colonization of mice (Liu *et al*., 2008). These studies and others (Cerdeno-Tarraga *et al*., 2005) illustrate the importance of DNA inversion as a mechanism to modulate gene expression and ultimately to increase survival in such environments.

At this stage we can only speculate on the advantages to *C. difficile* of expressing a phase-variable CWP. Expression of *cwpV* may be in response to a particular growth condition or environmental stimulus, but to date we have been unable to detect any conditions under which the frequency of DNA inversion or *cwpV* transcription is altered (Emerson *et al*., 2008 and data not shown). CwpV is the only CWP that is known to undergo phase variation in *C. difficile.* Analysis of the genome sequence of 630 did not reveal copies of the IRs associated with *cwpV* at any other locations in the genome. As recombinases are highly specific for their target nucleotide sequences, this suggests that *cwpV* is the only phase-variable gene in *C. difficile* regulated by RecV. Further analysis may of course reveal other genes regulated by DNA inversion mediated by different recombinases. Our ongoing work is concentrating on the detailed mechanisms underlying the control of *cwpV* expression as well as investigating the function of the CwpV protein.

It appears that the DNA inversion of the *cwpV* switch is controlled by one or more recombinases that are specific to *C. difficile*. The recombinase identified here, RecV, is present in all strains of *C. difficile* so far investigated (Stabler *et al*., 2006) implying that this control mechanism of CwpV expression may be universal. The transcription levels of *recV* are not seen to be altered by any environmental stress so far investigated (Emerson *et al*., 2008). The recombinase RecV was able to mediate DNA inversion in both the ON to OFF and OFF to ON orientations when expressed in *E. coli*, suggesting that if other factors are necessary for DNA inversion they are highly conserved. In *E. coli*, the *fim* switch is controlled by two recombinases, *fimB* and *fimE,* which are located in the *fim* locus (Klemm, 1986). Therefore, there could be other recombinases in *C. difficile* that function alongside RecV to control CwpV expression via control of the *cwpV* switch orientation. Construction of a *recV* knockout strain will shed light on this. However, genetic manipulation of *C. difficile* is still challenging and techniques for complementation of knockout mutants by insertion of a single chromosomal copy of a wild-type or mutant allele have not yet been described. Such experiments would be highly informative and would allow us to study the behaviour of a single copy of a mutated *cwpV* gene or promoter.

Most *C. difficile* CWPs are not members of the LPxTG protein family which are attached to the peptidoglycan via the sortase pathway (Marraffini *et al*., 2006), but are instead non-covalently anchored to the underlying cell wall by an as yet uncharacterized mechanism. The majority of predicted *C. difficile* CWPs, including CwpV, are members of a large family of CWPs which are localized to the cell wall via non-covalent interactions (Emerson and Fairweather, 2009). Other characterized members of this family include the S-layer proteins (SlpA), the adhesin Cwp66 and the protease Cwp84 (Waligora *et al*., 2001; Janoir *et al*., 2007). Our results show that CwpV is processed by internal cleavage to produce two proteins that are localized to the cell wall. We have recently described a similar mechanism for the two SLPs, HMW SLP and LMW SLP, which after cleavage re-associate via defined domains to produce a stable complex on the cell surface (Fagan *et al*., 2009). Whether CwpV is processed by the same machinery as that used for SlpA and whether the two CwpV cleavage products re-associate as a complex is unknown but is currently under investigation. We have shown that CwpV can constitute a large proportion of total cell surface protein and may therefore interact with SlpA in order to maintain the integrity of the S-layer. The level of CwpV expression in single ON cells is unknown but construction of a *recV* knockout strain will allow us to determine the level of expression from cells with the *cwpV* switch ON.

In this study we have shown that expression of the *C. difficile* cell wall protein CwpV is phase variable and controlled by a novel switch employing an intrinsic terminator. To our knowledge this is the first description of phase variation controlled by DNA inversion in Clostridia and it will be of interest to determine whether the mechanism described here is found in other members of the genus. The functional consequences of phase variation of CwpV, particularly in the context of pathogenesis, remain to be elucidated and are currently under investigation in our laboratory.

## Experimental procedures

### Bacterial strains and growth conditions

Bacterial strains and plasmids used in this study are described in Tables 1 and 2 respectively. *C. difficile* and *C. perfringens* were routinely cultured either on blood agar base II (Oxoid, Basingstoke, UK) supplemented with 7% defibrinated horse blood (TCS Biosciences, Botolph Claydon, UK), BHI agar (Oxoid) or in BHI broth (Oxoid). Cultures were grown in an anaerobic cabinet (Don Whitley Scientific, Shipley, UK) at 37°C in an atmosphere of 10% CO_2_, 10% H_2_ and 80% N_2_. *E. coli* was grown at 37°C on LB agar or in LB broth supplemented with appropriate antibiotics.

**Table 1:** Strains used in this study.

Strain	Toxin status	Ribotype	Characteristics/reference
*Clostridium difficile*			
630	A+, B+	012	Wust and Hardegger (1983)
630Δ*erm*	A+, B+	012	Hussain *et al*. (2005)
630Δ*erm*Δ*cwpV*	A+, B+	012	This study
CDKK371	+ve^a^	001	Calabi and Fairweather (2002)
Y	Non-toxic	010	Calabi *et al*. (2001)
R8366^b^	A+, B+	001	Calabi and Fairweather (2002)
R20291	A+, B+ CDT+	027	Stabler *et al*. (2006)
R20352	A+, B+ CDT+	027	Stabler *et al*. (2006)
CDKK167	+ve	016	C. Kelly
NF2029	A+, B+	106	M. Wilcox
R7404	A−, B+	017	J. Brazier
M9	A−, B+	017	D. Drudy
AY1	A−, B−	N.D.	D. Gerding
AY5	A−, B−	N.D.	D. Gerding
*Clostridium perfringens*			
SM101	*gusA*		B. Dupuy

a +ve, toxin A positive but toxin B unknown.

b No expression of *cwpV*. This strain was sourced from two independent labs. Neither isolate expressed *cwpV* and both contained the 2 bp deletion.

**Table:** Plasmids used in this study.

Name	Relevant characteristics	Source/reference
pMTL007	*E. coli-Clostridium* shuttle vector containing the Ll.ltrB group II intron with a retrotransposition-activated *ermB* cassette	Heap *et al*. (2007)
pIC007: Cdi-cwpV-1056s	pMTL007 with the Ll.ltrB intron retargeted to insert after base 1056 in *C. difficile* 630 *cwpV* ORF	This study
pTUM177	Contains promoterless *gusA* gene with *C. difficile tcdB* RBS	Mani and Dupuy (2001)
pMTL960	*E. coli – C. difficile* shuttle vector	Purdy *et al*. (2002)
pCBR023	Contains modified *gusA* gene under control of *cwp2* promoter; pUC19 backbone	This study
pCBR026	pUC19 containing *gusA* and long upstream sequence of *cwpV* with switch in ON orientation	This study
pCBR027	pUC19 containing *gusA* and long upstream sequence of *cwpV* with switch in OFF orientation	This study
pCBR034	Insert from pCBR023 cloned into pMTL960	This study
pCBR037	Insert from pCBR026 cloned into pMTL960	This study
pCBR038	Insert from pCBR027 cloned into pMTL960	This study
pCBR039	Derivative of pCBR037 containing 2 bp deletion in LIR (LIR*) of *cwpV* switch	This study
pCBR041	Derivative of pCBR037 containing 2 bp deletion in RIR (RIR*) of *cwpV* switch	This study
pCBR040	Derivative of pCBR038 containing 2 bp deletion in LIR (LIR*) of *cwpV* switch	This study
pCBR042	Derivative of pCBR038 containing 2 bp deletion in RIR (RIR*) of *cwpV* switch	This study
pCBR035^a^	pMTL960 containing *gusA* and short upstream sequence. *cwpV* switch in ON orientation	This study
pCBR036	pMTL960 containing *gusA* and short upstream sequence. *cwpV* switch in OFF orientation	This study
pCBR043	Derivative of pCBR037 with entire *cwpV* switch deleted	This study
pCBR044	pMTL960 containing the full-length *cwpV* gene from 630 under the control of *cwp2* promoter	This study
pCBR045	pACYC-Duet1 containing *CD1932*	This study
pCBR046	pACYC-Duet1 containing *CD1905*	This study
pCBR047	pACYC-Duet1 containing *CD2066*	This study
pCBR048	pACYC-Duet1 containing *CD1882*	This study
pCBR049	pACYC-Duet1 containing *CD1167* (*recV*)	This study
pCBR050	pACYC-Duet1 containing *CD3578*	This study
pCBR051	pACYC-Duet1 containing *CD1222*	This study

a pCBR035–043 have the pMTL960 backbone and were constructed by insertion of Acc65I-BamHI fragments from pCBR024 to pCBR032 which have a pUC19 backbone.

### DNA and RNA manipulations

DNA manipulations were carried out according to standard techniques (Sambrook *et al*., 1989). For use in DNA cloning, *C. difficile* genomic DNA was isolated as described previously (Calabi *et al*., 2001). PCRs used Taq polymerase (Sigma), Expand Long-Template Polymerase (Roche Diagnostics) or KOD (Merck) in accordance with the manufacturers' protocols. For PCR-RFLP the product of 40 rounds of PCR amplification were digested using *Ase*I (New Englad Biolabs). Details of plasmid construction including all primer sequences (Table S1) are given in the *Supporting information*.

### Conjugation into *C. difficile* and *C. perfringens*

Plasmids were transformed into *E. coli* CA434 and then conjugated into *C. difficile* 630 or *C. perfringens* SM101 as described by Purdy *et al*. (2002) using thiamphenicol (30 μg ml^−1^) to select for pMTL960-based plasmids and cycloserine (250 μg ml^−1^) to counter-select for *E. coli.*

### Construction of a *cwpV* knockout mutant in *C. difficile* 630

A *cwpV* mutant was generated in *C. difficile* 630Δ*erm* (Hussain *et al*., 2005) by insertion of a bacterial group II intron containing a retrotransposition-activated marker (RAM) conferring erythromycin resistance (Zhong *et al*., 2003) using the ClosTron clostridial gene knockout system (Heap *et al*., 2007). An Ll.ltrB target site was identified within the 630 *cwpV* gene and intron retargeting primers NF1003-NF1005 were designed using the online TargeTron algorithm (http://www.sigma-genosys.com/targetron/). Plasmid retargeting was carried out as previously described (Heap *et al*., 2007) and the resulting plasmid, pIC007:Cdi-*cwpV*-1056s was transferred to *C. difficile* 630 by conjugation from *E. coli* CA434. Four to five colonies of 630 (pIC007:Cdi-*cwpV*-1056s) were resuspended in 1 ml of anaerobic BHI with isopropyl-β-D-thiogalactopyranoside (IPTG) 1 mM and thiamphenicol (30 μg ml^−1^) and incubated at 37°C for 3 h. The bacteria were then harvested, resuspended in 1 ml of fresh BHI broth and incubated for a further 2 h. Potential Ll.ltrB insertions were selected by plating bacteria on BHI agar supplemented with erythromycin (2.5 μg ml^−1^). Following subculture, putative *cwpV* mutants were screened by colony PCR using primers specific for the inserted RAM (NF722 and NF723), the *cwpV* gene (NF1064 and NF1065) and each of the *cwpV* gene primers in combination with a primer internal to the Ll.ltrB intron (NF1063). A single *cwpV* mutant was further characterized by western immunoblotting using antibodies specific for CwpV (see below) and designated 630Δ*erm*Δ*cwpV*.

### β-Glucuronidase assays

β-Glucuronidase enzyme activity was measured in lysates of *C. difficile* cultures as described previously (Dupuy and Sonenshein, 1998; Mani *et al*., 2002) using the substrate p-nitrophenyl-β-D glucuronide (Sigma).

### Purification of recombinant *CwpV* protein and generation of antibodies

The first repeat of *cwpV* from *C. difficile* 630 (CwpVrpt1) was amplified by PCR using primers NF345 and NF346 and cloned into pGEX-4T-1. *E. coli* transformants were grown in LB to OD_600_ 0.4 and expression was induced by addition of 1 mM IPTG. Bacteria were harvested after 2 h, lysed by sonication and the fusion protein purified by glutathione affinity chromatography. The GST tag was cleaved using thrombin and the proteins separated by size exclusion chromatography. The purified CwpV repeat was inoculated into rabbits to raise antisera recognizing CwpVrpt1. Antibodies were affinity purified using a column of CwpVrpt1-coated Affi-gel 10 (Bio-Rad).

The conserved N-terminal 48.9 kDa of CwpV (CwpVNter) was cloned from *C. difficil*e 630 into pENTR-TEV-D-TOPO and subsequently subcloned to pDEST15 (Invitrogen). *E. coli* transformants were grown overnight at 25°C in Overnight Express media (Novagen/Merck Chemicals). Bacteria were harvested, lysed by sonication and the fusion protein purified by glutathione affinity chromatography followed by size exclusion chromatography. The purified protein was inoculated into rabbits to raise antisera recognizing CwpVNter. LMW SLPs were produced as described previously (Fagan *et al*., 2009) and antisera generated in rats. All antisera were generated commercially (Eurogentec).

### Immunodetection

#### 

##### 

###### Western immunoblotting

*C. difficile* were grown overnight in BHI broth and harvested by centrifugation. Glycine cell-surface extracts were performed as described previously (Calabi *et al*., 2001). Proteins were subjected to SDS-PAGE and Western blotting according to standard protocols. Anti-CwpVrpt1 and anti-CwpVNter were used at the dilutions indicated in the text, followed by anti-rabbit-HRP (Dako Cytomation) at 1/2000. Blots were developed using SuperSignal West Pico Chemiluminescent Substrate (Pierce).

###### Immunocytochemistry

*C. difficile* cells from liquid culture were washed with PBS then fixed in 8% formaldehyde in PBS, which was quenched using 20 mM NH_4_Cl for 15 min. The washed cell suspension was spotted onto a glass slide and allowed to dry. The bacteria were incubated with 1/10 rabbit anti-CwpVrpt1 and 1/20 rat anti-LMW-SLP, then washed and incubated with 1/10 anti-rabbit-FITC (Dako Cytomation) and 1/40 anti-rat-rhodamine red (Jackson ImmunoResearch Laboratories, West Grove, PA).

###### Flow cytometry

Ten microlitres of an overnight liquid culture of *C. difficile* was washed, resuspended in 1/10 rabbit anti-CwpVrpt1, washed and finally resuspended 1/10 anti-rabbit-FITC (all washes and dilutions in PBS). Cells were washed and resuspended in 200 μl PBS, and analysed using a Becton Dickinson FACS Calibur.

###### Immunogold electron microscopy

Colonies were scraped from blood agar plates and resuspended in cold 4% paraformaldehyde (PFA) and 0.2% glutaraldehyde in PBS, and were fixed on ice for 10 min. The cells were harvested, resuspended in 4% PFA and 0.2% glutaraldehyde and fixed for a further 1 h. The bacteria were washed three times in PBS, infiltrated with 2.3 M sucrose in PBS overnight at 4°C, attached to a specimen stub and plunge-frozen in liquid nitrogen. The 65 nm ultrathin cryosections were cut on a Leica UCT ultramicrotome with EM FCS cryoattachment. Sections were mounted on grids with formvar support and blocked with 0.02 M glycine in PBS followed by 10% FCS in PBS; this was the diluent for all subsequent steps. Sections were incubated in anti-CwpVrpt1 for 1 h followed by three 5 min PBS rinses. Sections were incubated in 15 nm Protein A gold for 30 min followed by three 5 min PBS rinses. Sections were rinsed in double distilled H_2_O six times over 5 min. The sections were then contrasted with uranyl acetate in methyl cellulose on ice for 10 min. The sections were air dried and viewed in a CM100 transmission cryo-electron microscope (Philips, Guildford, UK) at 16 000× to 32 000× magnification.
