# Supplementary material for: A novel genetic switch controls phase variable expression of CwpV, a Clostridium difficile cell wall protein
Source: Mol Microbiol. 2009 Aug 6;74(3):541–56. doi: 10.1111/j.1365-2958.2009.06812.x (PMC2784873; doi:10.1111/j.1365-2958.2009.06812.x)
Supplement: Supplementary file 1 [file mmi0074-0541-SD1.pdf]

SUPPLEMENTARY INFORMATION

A Novel Genetic Switch Controls Phase Variable Expression of CwpV, a *Clostridium difficile* Cell Wall Protein

Jenny E. Emerson, Catherine B. Reynolds, Robert P. Fagan, Helen A. Shaw, David Goulding and Neil F. Fairweather

Division of Cell and Molecular Biology, Imperial College London,
London SW7 2AZ, United Kingdom

n.fairweather@imperial.ac.uk; +44(0)20 7594 5247

CONSTRUCTION OF PLASMDS

gusA reporter plasmids

Plasmid pCBR001 was constructed (Codon Devices, USA) carrying a synthetic gene for the blue light photoreceptor FbFP (Drepper *et al.*, 2007) codon-optimsed for *C. difficile* and under the control of the constitutive cell wall protein Cwp2 promoter (P_{cwp2}) and its ribosome binding site (RBS). This plasmid was subsequently modified by the introduction of an *Eco*RI site 10 bp upstream of the RBS using primers NF812 and NF813 to create pCBR003. The *gusA* reporter plasmid pTUM177 (Mani and Dupuy, 2001), containing a promoterless *gusA* gene and the *tcdB* RBS, was mutated by deletion of the *Bam*HI site within *gusA* by SDM using primers NF883 and NF884. The *tcdB* RBS and the *gusA* ORF were amplified with primers NF885 and NF886, to produce an 1860 bp *Eco*RI - *Bam*HI fragment. This fragment was ligated to pCBR003 cleaved with the same enzymes to produce pCBR023. This was then cleaved with *Acc*65I - *Eco*RI and the P_{cwp2} fragment was replaced with one of the following fragments derived by PCR of *C. difficile* 630 DNA from upstream of *cwpV:* a short

fragment not including the putative *cwpV* promoter (P_{cwpV}) and having the *cwpV* switch on the ON orientation (primers NF814 and NF815) was used to form pCBR024; a short fragment not including the P_{cwpV} and having the *cwpV* switch on the OFF orientation (primers NF814 and NF815) forming pCBR025; a long fragment including the putative *cwpV* promoter was used with primers NF855 and NF856 and the transformants screened by PCR with the primers followed by *Ase*I digestion to identify a plasmid containing the *cwpV* switch in the ON orientation (pCBR026) and one in the OFF orientation (pCBR027). To produce pUC19-ON LIR*-GusA and pUC19-ON RIR*-GusA plasmids (pCBR028 and pCBR030) 2 bp deletions in the IRs of pCBR026 were carried out by inverse PCR. For ON LIR* primers NF864 and NF865 were used, for ON RIR* primers NF866 and NF867 were used. To produce pUC19-OFF LIR*-GusA and pUC19-OFF RIR*-GusA plasmids (pCBR029 and pCBR031) 2 bp deletions in the IRs of pCBR027 were carried out by inverse PCR. For OFF LIR* deletion primers NF864 and NF866 were used, for OFF RIR* production primers NF865 and NF867 were used. Inverse PCR was carried out with primers NF894 and NF895 to delete the entire *cwpV* switch from pCBR027 to form pCBR033. For plasmids pCBR023-032 described above, which are based on a pUC19 replicon, the *Bam*HI-*Acc*65I fragment containing the putative promoter sequences and the *gusA* gene were subcloned into pMTL960 to produce plasmids pCBR034-043.

CwpV over-expression plasmid (pCBR044)

CwpV was amplified from 630 gDNA using primers NF1096 and NF1097. This PCR product was digested with EcoRI and BamHI and cloned into pCBR023 in place of the *gusA* gene. The Acc65I-BamHI fragment containing PCwp2 and the cwpV gene was then subcloned into pMTL960 to produce pCBR044.

Recombinase expression plasmids

Genes for seven recombinase genes (CD1932, CD1905, CD2066, CD1822, CD1167, CD3578 and CD1222) were amplified from 630 gDNA using specific primers as detailed in Table S1. These PCR products were digested with BamHI and NotI and cloned into pACYCDuet-1 (Novagen) digested with the same enzymes.

References

Drepper, T., Eggert, T., Circolone, F., Heck, A., Krausz, U., Guterl, J.-K., Wendorff, M., Losi, A., Gartner, W., and Jaeger, K.-E. (2007) Reporter proteins for *in vivo* fluorescence without oxygen. *Nat Biotech* **25**: 443-445.

Mani, N., and Dupuy, B. (2001) Regulation of toxin synthesis in *Clostridium difficile* by an alternative RNA polymerase sigma factor. *Proc Natl Acad Sci U S A* **98**: 5844-5849.

Table S1. Primers used in this study.

Name	Sequence (5' to 3')	Characteristic or use
**NF1096**	CCGGAATTCAAATGGACAAGGGGGAAGAAAATAAAATG	Amplification of *cwpV* gene from 630 gDNA for cloning into pCBR023 to produce pCBR044
**NF1097**	CGCGGATCCCCAATACTAGTATTATCAAGAAAATTTACATC	Amplification of cwpV gene from 630 gDNA for cloning into pCBR023 to produce pCBR044
**NF345**	GCCGGATCCTCATCAAAGGTAGGTCAAATA	Cloning *cwpV* repeat for expression and raising antibody
**NF346**	CCGCTCGAGCTACACATATTTTTTAACTTCAAATGTTG	Cloning *cwpV* repeat for expression and raising antibody
**NF664**	CACCCAAACTGTGGCAACAAATTTAAC	Cloning *cwpV* CDS for expression of N-terminus and raising antibody
**NF665**	TTACTAAGAATTATCTCCCTTAACAC	Cloning *cwpV* CDS for expression of N-terminus and raising antibody
**NF654**	TGGTGAAAAAGGACTTGCAG	Amplification of *cwpV* cell wall binding region
**NF655**	CATCACCATTAGGCATGTTG	Amplification of *cwpV* cell wall binding region
**NF823**	GCATTTTTCCATCCATCTTG	Anneals at 5' end of *cwpV* gene, used to amplify for 5' RACE
**NF827**	GCAAATGGTGTTGCAGATAGAG	Amplification of *cwpV* upstream region for 5' RACE
**NF825**	TTTAAGGTAAGTTTGATTTTTATGTTAATGAAT	Anneals in inversion region
**NF826**	ATTCATTAACATAAAAATCAAACTTACCTTAAA	Anneals in inversion region
**NF824**	AAAAATATCGCAATTATTTGTTTTTCA	Anneals upstream of P*cwpV*
**NF863**	GTAAAAAGAGTAGCACTTTTAATTCTAAAGG	Anneals in left inverted repeat of the *cwpV* switch
**NF875**	TATAATGATTTCATAATTAATTTGTAATTATTTTGTTAC	Anneals in inversion region

Name	Sequence (5' to 3')	Characteristic or use
**NF812**	GGTAAATAAGGAAAAAATAATAAGAATTCGAATTTTTT AGGGGGAAAATACCATG	Introduction of *Eco*RI site 10 bp upstream of RBS in pCBR001
**NF813**	CATGGTATTTTCCCCCTAAAAAATTCGAATTCTTATTA TTTTTTCCTTATTTACC	Introduction of *Eco*RI site 10 bp upstream of RBS in pCBR001
**NF814**	GGTACCAATATCTAATTTTAAAGAGGTTTCTAACTATA ATG	Amplification of short ON from 630 gDNA
**NF815**	GAATTCACTTAATTTTTTAACTTTGTTCCCGTTCTAAT TCT	Amplification of short ON/OFF from 630 gDNA
**NF816**	GGTACCTTTTTATACAAAACCCTATTTTTTATAACAAT TC	Amplification of short OFF from 630 gDNA
**NF855**	CCGCCGGGTACCGTAATTTGAATTAAAATGTAAATAT AAAGAATAATTAAAAAAATATCGC	Amplification of ON/OFF from 630 gDNA
**NF856**	CCGCCGGAATTCACTTAATTTTTTAACTTTGTTCCCG TTCTAATTCT	Amplification of ON/OFF from 630 gDNA
**NF863**	GTAAAAAGAGTAGCACTTTTAATTCTAAAGG	Amplification of *cwpV* upstream region (Fig 5B; Fig 5D)
**NF864**	AAAGTGCTACTCTTTTTACAAATAAATTTTATTATTTAT ACTTG	Inverse PCR to delete 2 bp from IR
**NF865**	ATTCTAAAGGATACTTAATATCTAATTTTAAAGAGGTT TC	Inverse PCR to delete 2 bp from IR
**NF866**	ATTCTAAAGGCTACTTTTTTTATACAAAACCCTATTTT TTATAAC	Inverse PCR to delete 2 bp from IR
**NF867**	GAACGGGAACAAAGTTAAAAAATTAAGTGAATTCGAA TTTTTTAG	Inverse PCR to delete 2 bp from IR

Name	Sequence (5’ to 3’)	Characteristic or use
**NF883**	CTTTAACTATGCCGGGATACATCGCAGCGTAATGC	Site-directed mutagenesis to remove *Bam*HI site from *gusA*
**NF884**	GCATTACGCTGCGATGTATCCCGGCATAGTTAAAG	Site-directed mutagenesis to remove *Bam*HI site from *gusA*
**NF885**	CCGGAATTCCTGCAGTAAAGGAGAAAATTTTATGTTACG	Amplification of *gusA*
**NF886**	CGCGGATCCGGTGCGCCAGGAGAGTTGTTGATTC	Amplification of *gusA*
**NF894**	GTGCTACTCTTTTTACAAATAAATTTTATTATTTATACTTG	Inverse PCR deletion of region of inversion
**NF895**	CGGGAACAAAGTTAAAAAATTAAGTGAATTCGAATTTTTTAG	Inverse PCR deletion of region of inversion
**NF793**	CACCTCCTTTTTGACTTTAAGCCTACGAATACC	Anneals in pMTL960 backbone
**NF838**	CGCCAGCTGGCGAAAGG	Anneals in pUC19 backbone
**NF913**	CGCGGATCCAAATAATGATAAGAAAATTATAAAAGTACATAA	Amplification of recombinase CD1932 for cloning into pACYCDuet-1
**NF914**	ATAAGAATGCGGCCGCTTAATCGTCCTCAATCCATTCAA	Amplification of recombinase CD1932 for cloning into pACYCDuet-1
**NF915**	CGCGGATCCAAAAGCTGCAATTTATTCAAGA	Amplification of recombinase CD1905 for cloning into pACYCDuet-1
**NF916**	ATAAGAATGCGGCCGCCTAATTGCTTAAAGAAACAGAATTATC	Amplification of recombinase CD1905 for cloning into pACYCDuet-1
**NF917**	CGCGGATCCAAATGCATTTATTAGAAAAAGAAATAA	Amplification of recombinase CD2066 for cloning into pACYCDuet-1
**NF918**	ATAAGAATGCGGCCGCTTAATTAGCATTAAATAAACTTTCAGA	Amplification of recombinase CD2066 for cloning into pACYCDuet-1

Name	Sequence (5’ to 3’)	Characteristic or use
**NF919**	CGCGGATCCAAATGTGGCAATTTATTTACGCAAAA	Amplification of recombinase CD1882 for cloning into pACYCDuet-1
**NF920**	ATAAGAATGCGGCCGCTTAATCTCTTGGGATATTAGGGTAA	Amplification of recombinase CD1882 for cloning into pACYCDuet-1
**NF921**	CGCGGATCCAAAAATGGCAACAAGACCTATAGAAA	Amplification of recombinase CD1167 for cloning into pACYCDuet-1
**NF922**	ATAAGAATGCGGCCGCTTAACCAATAAAGAAATTTTCACTAGC	Amplification of recombinase CD1167 for cloning into pACYCDuet-1
**NF1043**	CGCGGATCCAATGATTATTATATATGGATATTGCCGTG	Amplification of recombinase CD3578 for cloning into pACYCDuet-1
**NF1044**	ATAAGAATGCGGCCGCCTAAATTGATTTTTTAGCACGAATAAGTGTGC	Amplification of recombinase CD3578 for cloning into pACYCDuet-1
**NF1045**	CGCGGATCCAATGGATATTATAGAGGGATATATAG	Amplification of recombinase CD1222 for cloning into pACYCDuet-1
**NF1046**	ATAAGAATGCGGCCGCCTATTTAGCTTCTGCTTTTTGTAATTCTAC	Amplification of recombinase CD1222 for cloning into pACYCDuet-1
**NF1003**	AAAAAAGCTTATAATTATCCTTAACTGCCAATGATGTGCGCCCAGATAGGGTG	IBS primer for retargeting the Ll.ltrB intron in pMTL007 to the 630 *cwpV* gene
**NF1004**	CAGATTGTACAAATGTGGTGATAACAGATAAGTCAATGATGTTAACTTACCTTTCTTTGT	EBS1d primer for retargeting the Ll.ltrB intron in pMTL007 to the 630 *cwpV* gene
**NF1005**	TGAACGCAAGTTTCTAATTTCGATTGCAGTTCGATAGAGGAAAGTGTCT	EBS2 primer for retargeting the Ll.ltrB intron in pMTL007 to the 630 *cwpV* gene
**NF1063**	CGAAATTAGAAACTTGCGTTCAGTAAAC	EBS universal primer
**NF722**	ACGCGTTATATTGATAAAAATAATAATAGTGGG	Amplification of *ermB*-RAM for screening of putative Ll.ltrB insertions

Name	Sequence (5' to 3')	Characteristic or use
**NF723**	ACGCGTGCGACTCATAGAATTATTTCCTCCCG	Amplification of *ermB*-RAM for screening of putative Ll.ltrB insertions
**NF1064**	CAAATAGTACAAGACTAGGTGGAACAG	Amplification of *cwpV* for screening of putative Ll.ltrB insertions
**NF1065**	GTACCTTTATTATCTATAGTAGTCCCTTTAG	Amplification of *cwpV* for screening of putative Ll.ltrB insertions
